# Delayed Retention of New Word-Forms Is Better in Children than Adults Regardless of Language Ability: A Factorial Two-Way Study

**DOI:** 10.1371/journal.pone.0037326

**Published:** 2012-05-16

**Authors:** Dorothy V. M. Bishop, Johanna G. Barry, Mervyn J. Hardiman

**Affiliations:** Department of Experimental Psychology, University of Oxford, Oxford, United Kingdom; Cuban Neuroscience Center, Cuba

## Abstract

**Background:**

Nonword repetition, the ability to retain and repeat unfamiliar sequences of phonemes is usually impaired in children with specific language impairment (SLI), but it is unclear whether this explains slow language learning. Traditional nonword repetition tests involve a single presentation of nonwords for immediate repetition. Here we considered whether rate of learning of novel phonological sequences was impaired when the same items were presented repeatedly.

**Methodology/Principal Findings:**

Three complex nonwords were each presented for repetition five times in two sessions (A and B) separated by one hour. We studied both adults and children from (i) families with a child with SLI and (ii) families whose children did not have SLI. This gave a 2×2 design with familial SLI as one factor, and age (up to or above 18 years) as the other. Overall, participants from families with SLI were poorer at nonword repetition than their peers from typical-language families, and there was a trend for children with SLI to show less within-session learning than typically developing children. However, between-session retention, measured as the difference between the last trial from session 1 and the first trial of session 2, showed a significant age effect, η^2^ = .139, p = .004, regardless of family SLI status. Adult participants showed a decrease in score from the last trial of session A to the first trial of session B, whereas children maintained their level of performance, regardless of whether or not they had SLI.

**Conclusions/Significance:**

Poor nonword repetition in SLI appears to reflect inadequate encoding of phonological information, rather than problems retaining encoded information. Furthermore, the nonword learning task is consistent with the notion of a sensitive period in language learning: Children show better retention over a delay for new phonological sequences than adults, regardless of overall level of language ability.

## Introduction

People vary in their language-learning ability; some have language-learning problems in the context of otherwise normal development and are diagnosed with specific language impairment (SLI), a strongly heritable condition affecting around 3–7% of children [1]. Studies of children with SLI have considered how far their deficits are specific to linguistic processing, and how far they may be secondary to general problems in perception or memory [2]. The notion of a core deficit in phonological short-term memory was first given prominence in a study by Gathercole and Baddeley [3], which found that children with SLI had severe problems in repeating nonwords. Subsequent studies have adopted a genetically informative design by including individuals with different degrees of genetic relationship to affected children and have identified impaired nonword repetition as a marker not only in children with SLI, but also in their first degree relatives [4], [5]. Nonword repetition has subsequently been used as a phenotype in molecular genetic studies searching for genes associated with SLI [6], [7].

Although poor nonword repetition is now well-established as a correlate of SLI [8], there remains some debate concerning the extent to which this impairment is responsible for language-learning problems. In early work, Gathercole and colleagues emphasised the importance of nonword repetition as an indicator of a phonological short-term memory. However, there are multiple factors that can influence performance, including phonological segmentation, encoding, and articulatory ability [9]. The relationship between nonword repetition and vocabulary learning has also been a topic of debate. Studies of young children suggested that it indexes a phonological memory system that is important for vocabulary learning [10]. However, subsequent work has queried this interpretation, and it has been suggested instead that positive correlations between these skills may reflect the fact that vocabulary knowledge restructures phonological representations [11]. Consistent with this, in typically-developing children, the relationship between nonword repetition and vocabulary weakens considerably by the age of eight years [12]. Furthermore, unaffected siblings of children with SLI often have impaired nonword repetition despite normal vocabulary skills [4].

If the language learning rate is limited in children with SLI by poor phonological memory, we would expect to see this when children's language learning is tested over time. In contrast to this prediction, Edwards and Lahey [13] found that the *rate* of learning was normal in children with SLI when they were repeatedly presented with novel phonological sequences, even though their overall repetition ability was impaired. From an analysis of error patterns and response latencies, they concluded that the problems with nonword repetition were not caused by poor auditory perception or motor execution, and were more likely due to the way in which new information was encoded in phonological memory, with reliance on holistic rather than segmented representations. This suggests, then, that the task indexes poor encoding of phonological information, rather than rapid decay in memory.

Edwards and Lahey did not, however, consider how well the newly-learned nonwords were retained. A study by Rice et al. [14] suggested that newly-learned information may be less well retained over a delay in children with SLI. They studied 4- to 5-year-olds and showed that although they showed evidence of incidental learning of new words presented ten times in the course of a video, they retained less information than age-matched controls when retested after 1–3 days. The mean score of the age-matched controls increased over the delay, suggesting consolidation in long-term memory, whereas it declined for the children with SLI.

In the current study, we wished to test whether short-term and long-term learning of new words is compromised in children with SLI. We tested the hypothesis that children with SLI and their adult relatives would show poor retention of phonological material over a delay. This hypothesis was not confirmed: instead, we found that age rather than familial SLI was a key factor. Regardless of SLI status, adults tended to show a decline in performance after a delay, whereas children did not.

## Materials and Methods

The study was approved by the Oxford Psychiatric Research Ethics Committee; parents of all participants gave written informed consent, and the children themselves gave assent after the study was explained in age-appropriate language.

### Participants

The study had a 2×2 design, with age (up to or above 18 years) as the first factor and familial SLI status as the second. Participants were drawn from a family study of SLI [5]. Both children and their adult first degree relatives (parents and older siblings) were included. The children with SLI had been recruited from special educational placements for children with language difficulties and all met research criteria for SLI by scoring 1 SD or more below the population mean on at least two language measures: see further details in Barry et al. [5]. The ‘typical language’ control participants came from families where the children were not diagnosed with SLI and did not meet SLI criteria on the language battery. For the current study, the pool of participants was subdivided into the four groups shown in Table 1, i.e. by age band (up to or above 18 years) and by family status. Those from SLI families were either children who had been identified with SLI, or their older siblings or parents.

**Table 1:** Gender, age and psychometric test scores of sample, divided by family status and age band.

Family status	Typical language				SLI			
Age band	Child		Adult		Child		Adult	
N female∶male	8∶6		8∶8		1∶10		8∶10	
	Mean	SD	Mean	SD	Mean	SD	Mean	SD
Age (yr)	12.4	(2.41)	42.9	(8.94)	14.6	(2.13)	43.1	(13.3)
Nonverbal IQ, ss	111.2	(14.16)	120.1	(10.23)	99.6	(10.65)	119.1	(12.09)
Scaled scores								
TOWRE sight word, ss	103.4	(12.47)	92.6	(13.57)	87.5	(16.52)	93.5	(14.07)
TOWRE phonological decoding, ss	107.0	(11.52)	104.4	(12.32)	86.6	(20.19)	92.9	(11.68)
Test for Reception of Grammar, ss	106.4	(8.21)	102.9	(6.35)	89.3	(16.98)	100.9	(7.15)
Raw scores								
NEPSY nonword repetition, raw/46*	38.4	(3.32)	39.4	(4.62)	29.6	(8.25)	36.4	(5.02)
TOWRE sight word, raw/104	71.4	(16.17)	88.4	(12.37)	57.0	(25.70)	87.8	(14.3)
TOWRE phonological decoding, raw/63	40.0	(13.52)	55.9	(6.14)	25.6	(20.80)	47.0	(9.94)
Test for Reception of Grammar, raw/20	17.8	(1.81)	18.7	(1.34)	14.2	(4.47)	18.3	(1.57)

* Data missing on this test for two of the Typical Adults.

### Psychometric screening

A short psychometric battery was administered to confirm that the typical-language families had normal range scores, and to quantify extent of language-literacy problems in the SLI family members. Participants were administered the block design and matrix reasoning task from the Wechsler Abbreviated Scale of Intelligence [15] to assess non-verbal reasoning skills. It was not possible explicitly to match groups on nonverbal ability, given the constraints imposed by testing whole families, but individuals were included only if they had nonverbal IQ of 85 or above. The electronic version of the Test for Reception of Grammar-2 [16] was used to assess receptive grammatical knowledge. Raw scores (number of blocks correct) were converted into scaled scores using norms derived from British adults. Reading skills were assessed using the Sight Word Efficiency and Phonemic Decoding Efficiency subtests of the Test Of Word Reading Efficiency [17]. Raw scores were converted to scaled scores using American norms for adult readers.

The standard NEPSY nonword repetition test [18] was given after the experimental nonword learning task (see below), but with the three test nonwords excluded. The first trial of the experimental learning session was used to score these three nonwords for the NEPSY. Because the NEPSY nonword repetition test has norms only up to 12 years of age, raw scores are presented here.

### Experimental nonword learning

We designed a task where the same three polysyllabic nonwords were presented for repetition by the participant five times in succession. The nonwords were then re-presented a further five times after a one-hour break. This meant we could look at learning both within a session (over the five trials for each item) and between trials.

The three nonwords were selected from the NEPSY nonword repetition subtest [18] on the basis that previous studies had indicated they were especially difficult, even for adults, to repeat. Recordings of these nonwords, ‘pres**krim**skee’ (3 syllables, duration 1030 ms), ‘craspre**scrine**ter’ (4 syllables, duration 1690 ms), and ‘**skri**fluna**fliss**trop’ (5 syllables, duration 1920 ms), were made by an adult female with a southern English accent. Stress was placed on the bolded syllables. (N.B. Due to a mistake in the pronunciation, the ‘t’ phoneme was omitted from the end of the first syllable of the second nonword in the list).

Each participant received the instruction: “In a moment I would like you to put on some headphones and I will play you three series of words. You won't have heard any of the words before and some may sound a little odd. I want you to listen very carefully to each word and then repeat exactly what you hear.”

In the first session (session A), each nonword was presented five times in a row with a fixed gap of ten seconds between each presentation. The participant immediately repeated each nonword after it was presented. The sequence of presentation was always 3, 4 and 5 syllable nonword. Exactly the same procedure was repeated as close as possible to, but never less than, one hour later for session B. During the interval, participants either relaxed in a waiting area where snacks, toys, DVDs and reading materials were present, or underwent other cognitive tests, or underwent a brain scan. Members of a family were tested on the same day, and there were no systematic differences in the activities that adults and children did in the interval. The nonword learning task was scored in terms of number of syllables correct on each trial based on an initial broad phonetic transcription. This was performed immediately online, and checked subsequently from the audiorecording. For a syllable to be judged correct, all consonants and vowels in the heard syllable had to be present in the uttered syllable, in the right order and without the insertion of any additional phonemes. Very few errors on vowels were observed. Most errors made involved consonant clusters, with errors ranging from consonant transpositions, insertions, or substitutions. Consistency of scoring was checked by comparing two independent scorings of 100 trials; this gave an intraclass correlation of .83.

## Results

Table 1 shows raw scores as well as scaled scores for language and literacy measures, to facilitate comparisons of absolute level of performance, regardless of age. Note that the raw score of the adults from the SLI families did not differ significantly from that of the children from Typical-language families on any measures except TOWRE sight words, where the adults were superior.

**Figure 1:**
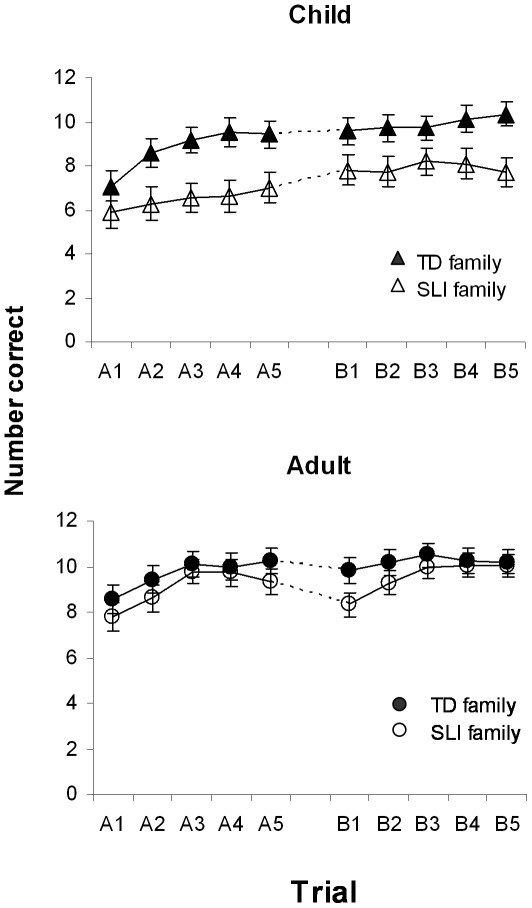
Mean number of syllables correct on each trial for each of two sessions of nonword learning task in relation to family status and age band. Error bars show SE. Max score = 12.

Mean number of syllables correct for the four groups are shown in Figure 1. The numbers of syllables correct on each trial for each session were transformed into arcsin proportion scores, to reduce skew, and entered into a four way analysis of variance, with Family status (Typical language or SLI) and Age band as between-subjects factor, and Session and Trial as repeated measures. Greenhouse-Geisser correction was applied to correct for violation of sphericity affecting the Trial term. Analysis output in Table 2 shows that in addition to significant effects of all main terms, there is a significant interaction between Session and Age band. Post-hoc t-tests on raw scores showed that both Age bands showed evidence of learning, with improved scores from session 1 to 2 (children, mean session 1 = 7.74, SD = 2.77; mean session 2 = 9.02, SD = 2.61, t = 6.7, DF = 24, p<.001; adults, mean session 1 = 9.34, SD = 1.84; mean session 2 = 9.84, SD = 1.71, p = .001). T-test also confirmed that the extent of improvement was significantly greater for children than for adults, t = 3.46, DF = 57, p = .001.

**Table 2:** Output from 4-way ANOVA with factors Family status, Age band, Session and Trial.

Between-subject effects	F	Sig.	partial η^2^
Family status (Fam)	4.7	.034	0.079
Age band (Age)	4.5	.039	0.075
**Within-subject effects**			
Session	41.6	<.001	0.431
Session×Fam	0	.863	0.001
Session×Age	8.8	.005	0.138
Session×Fam×Age	0	.862	0.001
Trial	21.0	<.001	0.277
Trial×Fam	1.0	.393	0.018
Trial×Age	2.6	.065	0.044
Trial×Fam×Age	3.2	.029	0.056
Session×Trial	2.2	.086	0.038
Session×Trial×Fam	1.6	.178	0.029
Session×Trial×Age	0.1	.978	0.001
Session×Frequency×Group	2.0	.112	0.035

We had anticipated that those from SLI families may forget more during the one hour delay, but scrutiny of Figure 1 shows a very different pattern. Rather, the scores of adults appear to decline between the last trial of Session A and the first trial of Session B, in contrast to an increase for the child participants. To confirm this impression, a difference score between trial A5 and B1 was computed for each participant, and entered in a two-way ANOVA. Means for child Typical, child SLI, adult Typical and adult SLI were 0.14 (SD = 1.52), 0.82 (SD = 2.04), −0.44 (SD = 0.81) and −1.00 (SD = 1.64) respectively, confirming that scores in general increased for the child participants and decreased for adults, regardless of family SLI status. For difference scores, the main effect of Age band was significant, F (1, 55) = 8.87, p = .004, η^2^ = .139, whereas the main effect of Family status was not, F (1, 55) = 0.20, p = .889, η^2^ = 0. The interaction between these factors was also nonsignificant, F (1, 55) = 2.36, p = .130, η^2^ = .041. In a further analysis, one-sample t-tests were used to consider whether the change scores differed from zero. The gains in scores for the children were not reliably different from zero, mean = 0.44 (SD = 1.75), t (24) = 1.25, p = .223; the decrements in the adult groups were significant, mean = −0.74 (SD = 1.33), t (33) = −3.22, p = .003.

It could be argued that the Age band effect might be a consequence of superior nonword repetition ability in adults overall, which might limit the extent to which they could improve. As it turned out, it was possible to address this question by a comparison of child Typical and adult SLI groups. As noted earlier, these two groups were closely similar on the measures shown in Table 1. As evident from Figure 1, they also achieved similar levels of performance on the last trial of session A, with a mean arcsin proportion of 0.96 (SD = 0.29) for the child Typical group and mean of 0.99 (SD = .39) for the adult SLI group, t (29.9) = 0.33, p = .743. However, in terms of the A5-B1 difference score, the groups diverged, with the child Typical group having a mean of 0.14 (SD = 1.51), and the adult SLI group a mean of −1.0 (SD = 1.65), t (29.1) = 2.04, p = .05. Thus even though the adult SLI group was several years older than the child Typical group, and equivalent in performance at the end of session A, they retained less over the delay.

As shown in Table 2, the three-way interaction between Trial×Family status×Age band was also significant. This was explored further by taking the slope of the function for the average increase from trials 1 to 5 for each individual as a measure of within-session learning, and comparing the four groups on this measure with a one-way ANOVA. The main effect of group fell short of significance, though there was a non-significant trend for a shallower slope in the child SLI group (mean slope = 0.14, SD = 0.27) than in the other three groups: mean for child Typical = 0.38, SD = 0. 35; mean for adult SLI = 0.42, SD = 0.39; mean for adult Typical = 0.24, SD = 0.25; F (3, 55) = 2.21, p = .098. A total sample of 120 (twice that used here) would be needed to give power of .80 to identify this size of effect [19]. In a final analysis exploring the source of the interaction, one-way Anovas were run comparing the four groups on mean score for each trial, using LSD tests set at .05 level for post hoc comparisons. Consistent with the analysis of slopes, it was found that on trial 1, the child SLI group differed significantly only from the adult Typical group, but on all subsequent trials, they obtained lower scores than all three other groups.

## Discussion

This study considered whether individuals with SLI and their relatives were impaired at learning novel words, both within and between sessions. Consistent with Edwards and Lahey [13], we found that children with SLI did poorly overall in nonword repetition. Poorer performance compared to adult controls was also found for their parents and older sibling. For children with SLI there was a trend for slower within-session learning, but this was not seen in their parents and older siblings. Contrary to prediction, and encouraging to those attempting intervention with these children, we found good retention of material between sessions in children with SLI; despite their low levels of performance, their repetition scores were slightly (though nonsignificantly) higher after a one hour delay. This was a surprising result, which supports the interpretation of nonword repetition deficits as an indicator of poor phonological encoding, rather than rapid decay of information in memory - see also Barry et al. [20], which presents electrophysiological evidence further supporting this interpretation.

The question arises as to why our findings differ from those of Rice et al. [14], who found gains in remembering newly-learned words after a delay in typical children but not in those with SLI. There are many differences between the studies that could be responsible: the children in the Rice et al study were younger than those studied here and all had poor receptive vocabulary. The sample was also larger, giving more power to detect small effects. In addition, memory was assessed 1–3 days after initial learning, whereas our study used a delay of just one hour. Furthermore, Rice et al. used an incidental learning paradigm where novel words were presented in a meaningful context, and mastery of the novel words was tested by a multiple choice comprehension test; this contrasts with the current study where novel words were presented without meanings and the measure of mastery was accuracy of repetition.

Our study was not, however, insensitive to differences in delayed memory: In contrast to SLI status, age had a significant effect on nonword learning between sessions. This effect was seen when nonwords were re-presented after a delay of around one hour. We would not have been surprised if the adults had shown *greater* gains than child participants, given that adults tend to do better on a wide range of cognitive tasks, including auditory perceptual learning [21]. The surprising finding was that scores of the child group showed a non-significant trend for improvement over the delay, whereas those of adults declined significantly. An obvious question is whether the adult decline might be due to a ceiling effect: maybe they simply had less room for improvement. To address this we made use of the serendipitous finding that on the last trial of Session A, adults from SLI families had scores that were similar to child participants from Typical families (see Figure 1), yet the scores of these two groups diverged markedly on the first trial of Session B.

Our design does not allow us to rule out the possibility that the decline in adult scores might have been seen had we administered a sixth trial immediately after the five trials from session 1, with no delay. However, there seems no a priori reason to predict such a dip specifically after five trials have been administered, whereas forgetting over an interval is consistent with many other memory phenomena.

As Ferman and Karni [22] have noted, few studies have compared language-learning by children and adults under equivalent conditions. In their study, they compared children and adults learning an artificial grammar rule, and concluded that adults were superior to children - the opposite of what was found in our study. There is, however, a potential confound. The adults started from a higher baseline. Ideally, to establish whether there was an age-dependent sensitive period, we need to compare younger and older learners who are comparable on initial level of performance. Our design made it possible to do this by comparing adults who have relatively poor language skills (from the SLI families) with children who have relatively good language skills (from the typical families). As shown in Table 1, these two groups were broadly comparable in raw scores on most language measures.

It is also worth noting that, though Ferman and Karni [22] found overall superior learning of an artificial grammar rule in adults compared to children, their reaction time data showed greater *between-session* gains in children than adults, and greater *within-session* gains in adult than children. This is compatible with findings in the current study indicating superior delayed memory in children.

The fact that the age bands differed regardless of the language level of the participants fits an explanation in terms of neuroplasticity, i.e. superior language learning capacity in the immature brain [23]. There has been much discussion of whether humans have sensitive periods for language learning, but the evidence is often ambiguous, because of confounding factors of prior learning. Thus we know that it is easier to attain native-like competence in a second language if it is learned in childhood than in adulthood [24], [25]. However, interpretation of such evidence is complicated because there may be interference effects between first and second languages. Interference effects are less likely to apply here, where all participants are exposed to a small set of novel but phonotactically legal sequences.

The data reported here suggest that after an initial exposure period, new phonological sequences are remembered by children, but tend to decay in adults, regardless of the initial language level of the participants. Results must be interpreted cautiously, given the relatively small sample sizes. Nevertheless, these data raise the intriguing possibility that these differences reflect biological differences in learning capacity between children and adults. In cats and rodents, for instance, age-dependent neurotransmitter levels affect visual learning [26] and in songbirds, where sensitive periods have been clearly shown, vocal learning is determined by differences in gene expression between juvenile and adult birds [27].

We conclude by noting the advantages of the methodology adopted here, of comparing adults and children of different ability levels. This not only gives information on how individual differences in ability affect performance, but also allows us to vary age while matching on ability level. This kind of ‘mental age match’ has been widely used in the study of developmental disorders, but has not hitherto been used in studies of neuroplasticity.
